# Social affiliation motives modulate spontaneous learning in Williams syndrome but not in autism

**DOI:** 10.1186/s13229-016-0101-0

**Published:** 2016-09-07

**Authors:** Giacomo Vivanti, Darren R. Hocking, Peter Fanning, Cheryl Dissanayake

**Affiliations:** 1 A.J. Drexel Autism Institute, Drexel University, 3020 Market Street, Suite 560, Philadelphia, PA 19104-3734 USA; 2 Olga Tennison Autism Research Centre, School of Psychology and Public Health, La Trobe University, Melbourne, VIC 3086 Australia; 3 Developmental Neuromotor and Cognition Lab, School of Psychology and Public Health, La Trobe University, Melbourne, Australia

**Keywords:** Social learning, Learning, Imitation, Social cognition, Autism, Williams syndrome

## Abstract

**Background:**

Children with autism spectrum disorder (ASD) and those with Williams syndrome (WS) have difficulties with learning, though the nature of these remains unclear.

**Methods:**

In this study, we used novel eye-tracking and behavioral paradigms to measure how 36 preschoolers with ASD and 21 age- and IQ-matched peers with WS attend to and learn novel behaviors (1) from the outcomes of their own actions (non-social learning), (2) through imitation of others’ actions (social learning), and across situations in which imitative learning served either an instrumental function or fulfilled social affiliation motives.

**Results:**

The two groups demonstrated similar abilities to learn from the consequences of their own actions and to imitate new actions that were instrumental to the achievement of a tangible goal. Children with WS, unlike those with ASD, increased their attention and imitative learning performance when the model acted in a socially engaging manner.

**Conclusions:**

Learning abnormalities in ASD appear to be linked to the social rather than instrumental dimensions of learning.

## Background

The link between social engagement and early learning processes has been subject to extensive theoretical and empirical investigation, dating back to Piaget’s [1] and Vygotsky’s [2] accounts to contemporary scholarship [3, 4]. Findings to date suggest that young children actively create and respond to learning opportunities in their environment using two fundamental mechanisms: social learning (learning from the social environment via observation and imitation of others’ behavior) and non-social learning (learning from the physical environment via trial and error) [5, 6]. The key difference between these two mechanisms concerns the role played by social engagement in the acquisition of knowledge [7]. For example, through social learning, a child can learn how to operate a toy by observing and imitating an adult during a social exchange. Through non-social learning, the child can learn from the consequences of his own different attempts to operate the toy, in the absence of interaction with knowledgeable others.

While non-social learning via trial and error enables the acquisition of knowledge through active experiments in the physical world [8], social learning affords the opportunity to (1) acquire knowledge relevant to instrumental goals (the *instrumental* function of imitative learning) and (2) align one’s own behavior to the model’s behavior, a phenomenon that promotes feelings of social connectedness and affiliation (the *social* function of imitative learning) [4, 9]. For example, the degree of social closeness between imitator and demonstrator influences how closely and how frequently typically developing children imitate others [10–12].

Early engagement in object and social interactions that afford learning experiences has been linked to later cognitive outcomes [13, 14]. Importantly, as different learning mechanisms and learning functions place different weight on social engagement, it is plausible that variations in sociability during early development may uniquely impact social dimensions of learning.

Autism spectrum disorder (ASD) and Williams syndrome (WS) provide a striking test case to investigate this link. From infancy, children with ASD and those with WS share some overlapping impairments in the use of gesture, pointing, and joint attention [15, 16]. However, they present with opposing profiles in their *motivation* for social engagement, which is atypically low in ASD [17] and atypically high in WS [18]. It remains unclear how these atypical social interaction styles affect the different social and non-social dimensions of learning. Understanding the nature of the learning difficulties in these two populations has therefore important implications for theoretical perspectives elucidating the link between social engagement and different types of learning. It will also have concomitant clinical implications in informing approaches to facilitate learning.

### Social and non-social learning in autism

Autism spectrum disorder (estimated prevalence 1:68, [19]) is a neurodevelopmental disorder characterized by early emerging disruptions in social communication and restricted/repetitive behaviors. Social learning via imitation in this population has been subject to extensive analysis, indicating that imitation appears to be impaired in children with ASD [20, 21]. Research in this area has linked atypical imitation in ASD to impaired visual-motor integration [22], abnormalities in visual attention to the model [23–25], failure to map observed actions onto motor plans for similar actions [26], atypical processing of social cues that modulate imitation in typical development [27, 28], and reduced social “drive” to imitate others [29]. It is still unclear whether imitation abnormalities are social in nature, reflecting the pathognomonic social impairment that characterizes ASD, or whether they are the expressions of learning difficulties independent of social context [30, 31]. Recent research indicates that trial and error learning might also be impaired in ASD, although evidence in this area is more limited [32].

### Social and non-social learning in Williams syndrome

Williams syndrome is a rare neurodevelopmental disorder (estimated prevalence of 1:7500 to 1:20,000 [33]) presenting with impaired visual-spatial abilities and social-pragmatic skills alongside an increased drive for social approach [18]. Children with WS appear to perform poorly in learning and imitation tasks taxing visual-motor skills [34, 35]. However, surprisingly limited work has been conducted on the social dimension of learning in this population. A recent study reported preliminary evidence that young adults with WS required more attempts to learn a new task compared to mental age-matched typically developing controls in both an imitation condition and a trial-and-error condition [36], suggesting that both social and non-social learning might be impaired in WS. However, Fidler and colleagues [37] documented enhanced imitation of facial expressions of emotions in children with WS. These findings leave open the question of how the unique social phenotype in WS affects development of social and non-social learning propensities and abilities.

Although the distinctive drive for socialization that characterizes WS has the potential to facilitate social learning, it is also possible that an excessive fascination for people is detrimental, rather than beneficial, to the ability to process information relevant for social learning. For example, difficulties in disengaging attention from faces (especially the eye region) in WS could disrupt the processing of other important cues provided by the model [38]. However, the role of social drive as a mediating factor in social/imitative learning remains largely unexplored.

### Aim in the current study

In this study, we aimed to examine visual attention and learning performance of preschoolers with ASD and WS across four experiments, with experimental paradigms involving social versus non-social learning and where imitating a model served instrumental functions versus social-affiliative motives. We employed, for the first time, an eye-tracking paradigm that permitted a direct examination of the link between atypical attention deployment and imitation performance in young children with ASD and WS. Manipulating the weight of social components across learning tasks and comparing populations presenting with contrasting profiles of social motivation afforded a unique opportunity to evaluate whether differences in drive for social engagement result in different learning styles in children with ASD and WS. We hypothesized that while the groups would perform similarly in tasks tapping non-social, instrumental dimensions of learning, the WS group would show superior performance in those based on social processes and motives.

## Methods

### Participants

The participants were 36 preschoolers with autism spectrum disorder (ASD; mean age = 45.5 months, SD = 11.2 months, range = 29.2–74.1) and 21 peers with William syndrome (WS; mean age = 52.5 months, SD = 17.2 months, range = 26.7–78.8) who participated in four experiments. Participants with ASD were recruited through the Victorian Autism Specific Early Learning and Care Center, an autism intervention program located at the La Trobe University Community Children’s Centre. Participants in the WS group were recruited through the Williams Syndrome Family Support Group (Victoria) and the Williams Syndrome Association Australia.

The diagnoses of ASD were previously made by community-based healthcare professionals and confirmed for the study using the Autism Diagnostic Observation Schedule (ADOS 2, [39]) administered by a clinician with demonstrated research reliability in the use of this measure. Exclusion criteria for the ASD group included the presence of uncorrected hearing or vision impairment, and the presence of a major medical problem. All participants with WS had their diagnosis confirmed with the positive fluorescent in situ hybridization (FISH) test and displayed the typical ~1.6-Mb heterozygous microdeletion at 7q11.23 [40].

Participants’ cognitive level was measured with the Mullen Scales of Early Learning (MSEL). Following [41] and [42], developmental quotient (DQ) scores were calculated for each MSEL subscale according to the formula: DQ = age equivalent scores/chronological age × 100 and averaged to create an overall developmental quotient. Additionally, participants’ adaptive behavior was assessed using the Vineland Adaptive Behavior Scales (VABS; [43]). The ASD and WS groups did not differ on chronological age, overall cognitive level, and overall adaptive behavior (see Table 1). However, as expected, children with WS had superior scores in the socialization subscale of the VABS.

**Table 1:** Participant characteristics

	ASD (*N* = 36)	WS (*N* = 21)	*t* test *p* value
Gender, M, F	34, 2	11,10	–
Chron. age (months): M (SD)	45.53 (11.21)	52.55 (17.22)	.10
MSEL visual reception AE: M (SD)	26.74 (11.37)	29.17 (16.29)	.57
MSEL fine motor AE: M (SD)	25.35 (8.63)	24.83 (10.10)	.85
MSEL receptive lang. AE: M (SD)	20.38 (10.30)	27.67 (14.60)	.07
MSEL expressive lang. AE: M (SD)	23.38 (10.65)	26.61 (13.69)	.39
MSEL visual reception T: M (SD)	30.12 (10.10)	24.13 (7.15)	.22
MSEL fine motor T: M (SD)	28.18 (13.42)	21.86 (3.68)	.08
MSEL receptive lang. T: M (SD)	28.03 (14.41)	25.47 (9.53)	.53
MSEL expressive lang. T: M (SD)	30.27 (15.77)	24.00 (7.24)	.15
MSEL total DQ: M (SD)	62.93 (27.99)	56.44 (16.88)	.34
VABS communication: M (SD)	71.81 (18.98)	70.57 (11.65)	.79
VABS daily living skills: M (SD)	72.59 (27.20)	70.43 (11.43)	.73
VABS socialization: M (SD)	72.50 (14.69)	80.81 (12.53)	.03
VABS motor skills: M (SD)	75.09 (19.32)	68.86 (11.00)	.14
VABS composite: M (SD)	69.81 (19.14)	69.86 (10.08)	.99
ADOS severity score: M (SD)	7.45 (1.88)		

*ASD* autism spectrum disorder, *WS* Williams syndrome, *MSEL* Mullen Scales of Early Learning, *VABS* Vineland Adaptive Behavior Scales, *Second Edition*; *ADOS* Autism Diagnostic Observation Schedule, *AE* Age Equivalent, *T* T score

### Procedure

The study was approved by the La Trobe University Human Ethics Committee and informed consent was obtained from the children’s parents.

The children were tested in a quiet room in one of three University or early intervention settings, depending on where the child was recruited. Three children with WS were administered the standardized tests and the experimental battery in their home due to traveling difficulties on the part of their families. The length of experimental testing was approximately 25 min. The experiments presented here were part of a larger study examining social and non-social learning in young children with ASD and WS.

#### Preliminary validation

The validation procedure for the tasks used in the battery involved a piloting stage where 20 typically developing (TD) preschoolers were administered preliminary and current versions of each of the experimental tasks. As these were not matched for mental age, motor skills and other variables relevant to performance in the experimental tasks, they were not included as a control group, but their performance was examined to test the validity of the experimental paradigm. It was found that tasks intended to measure spontaneous imitation did indeed elicit spontaneous imitation in 85 to 100 % of the TD group. Similarly, 95 % of children showed evidence of trial-and-error learning in the task intended to measure trial-and-error learning. Additionally, we found that spontaneous imitation in the whole sample was highly correlated (*r* = .74, *p* < .001) to scores on the VABS items addressing imitation (items 9, 12, 16, and 21 in the socialization - interpersonal relationships subscale).

#### Experiment 1

The aim in experiment 1 was to investigate social learning in preschoolers with ASD and WS in a task in which imitation served a social affiliation function. To this end, we measured participants’ spontaneous imitation in response to a playful, socially engaging model and to a “neutral” model performing arbitrary (non-instrumental) actions on objects. This paradigm, based on behavioral research with typically developing children by Nielsen and colleagues [10, 11] was developed to capture children’s propensity to imitate for the purpose of establishing social connectedness with the model. We hypothesized that spontaneous imitation would be modulated by the socially engaging behavior of the model in children with WS, but not in those with ASD.

##### Method

Children were invited to sit on a mat opposite two experimenters and were presented with a set of eight pairs of identical objects. There were four trials, involving four different sets of objects. Each trial comprised a “playful condition” and a “neutral condition.” In the playful condition, one of the two experimenters, after obtaining the child’s attention, performed an arbitrary action on one of the objects (e.g., swinging a toy-policeman like a pendulum, pushing a ribbon through the closed hand) in a playful, socially engaging way, which included emotional expressions of surprise and happiness, as well as playful and animated body language. After the demonstration, the experimenter put the object back on the floor together with the other objects, without giving any instructions. In the neutral condition, the second experimenter demonstrated another action on a different object using the same procedure, but in a “neutral” manner, i.e., without displaying facial or bodily emotions. In both conditions, the experimenter alternated gaze between the toy and the child. The sequence order of the playful and neutral conditions was counterbalanced across participants according to a fixed random order. The spontaneous behavior of the child in response to the “playful” and “neutral” demonstrations was recorded for later coding.

The coding procedure was based on a simple three-point Likert system, with participants receiving a 2 if they imitated the action performed by the model, a 1 if they acted on the same object used by the model, and a 0 for any other response (e.g., picking up a different object or not using any object). A total score was calculated by summing the performance scores for each item and then converting the sum into a percentage score. Therefore, participants would obtain a score of 100 if they imitated all the demonstrated actions and a score of 0 if they imitated none. Coding of the video-recorded sessions was conducted by two research assistants who were blind to group membership and study hypotheses. Interrater reliability between the two research assistants for the coding of performance data was calculated on 20 % of the entire data set, with a high intraclass correlation (.98). Three participants in the WS group were excluded due to technical error during video-recording of the experiment, which resulted in unusable video footage.

##### Results

**Fig. 1:**
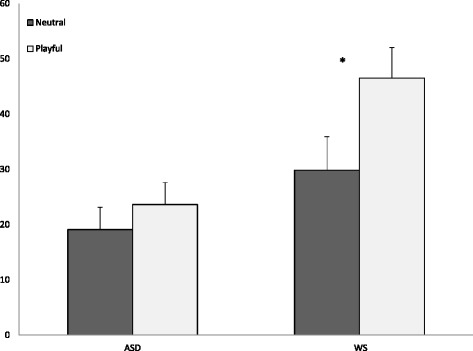
Imitation performance (proportion of imitated actions) in experiment 1 *p = .001

Imitation performance was analyzed with a 2 (group) × 2 (condition) ANOVA. There was a main effect of the group (*F* (1, 52) = 6.58, *p* = .01, *η*_p_2 = .11), a main effect of the condition (*F* (1, 52) = 18.63, *p* < .001, *η*_p_2 = .26), and a group × condition interaction (*F* (1, 52) = 5.46, *p* = .01. *η*_p_2 = .10). As illustrated in Fig. 1, pair-wise comparisons showed that while participants in the WS group imitated more in the playful/socially engaging condition compared with the neutral condition (adjusted *p* [Bonferroni] < .001, *η*_p_2 = .25), this was not the case in the ASD group (adjusted *p* = .12, *η*_p_2 = .04). Moreover, frequency of imitation was similar across groups in the neutral condition (adjusted *p* [Bonferroni] = .14, *η*_p_2 = .04), while in the playful/socially engaging condition, the WS group imitated significantly more than the ASD group (adjusted *p* = .001, *η*_p_2 = .18). While the majority of children across groups spontaneously imitated at least one action, 13 % of children in the ASD group and 11 % of children in the WS group did not engage in any imitative behavior. Results are summarized in Table 3.

In the ASD group, we found no associations between imitative performance and age, cognitive, adaptive, language, and motor functioning level (as assessed through the MSEL and VABS). Imitation of the playful model was negatively associated with the ADOS social affect calibrated social scores (*r* = −.43, *p* < .01), suggesting that children with more severe ASD symptoms in the social domain were less likely to imitate in response to the playful model. Conversely, there was no association between social symptoms and imitation of the neutral model. In the WS group, the only significant correlation was between imitation performance across conditions and chronological age (*r* = .55, *p* = .01 for imitation of the playful model and *r* = .73, *p* < .001 for the imitation of the neutral model), suggesting that older children in this group were more likely to engage in imitative behavior across conditions.

#### Experiment 2

In experiment 2, we used a novel eye-tracking paradigm to measure how participants’ visual attention to the model was affected by the presence/absence of socially engaging behavior. A similar procedure as in experiment 1 was used, expect that the “socially engaging” and the neutral model were presented through pre-recorded videos displayed on an eye-tracking computer to allow for analyses of visual attention. We hypothesized that visual attention and imitative response to the model would be modulated by the socially engaging behavior displayed by the model in the WS group, but not in the ASD group.

##### Methods

**Fig. 2:**
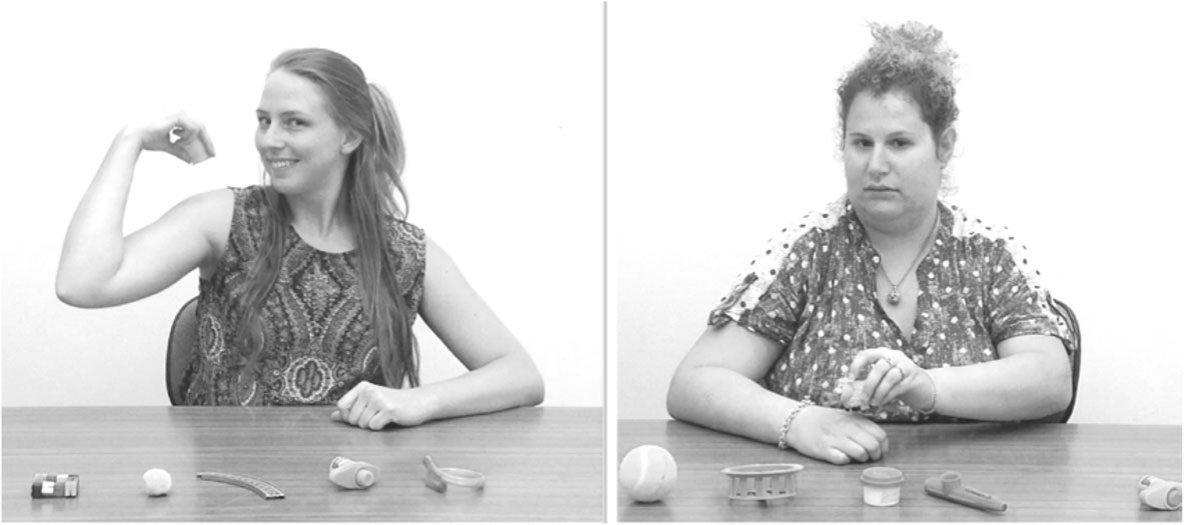
Playful model (*left*) and the neutral model (*right*) in experiment 2 video stimuli

Children were shown a series of eight video stimuli (10 s each) on a Tobii T120 binocular eye-tracker monitor with an imbedded camera (120 Hz, 1280 × 1024 pixel resolution, average precision of 0.5 of visual angle). In each video, a female demonstrator performed a simple action involving one of eight objects placed on the table in front of her. Examples of the actions used include moving a slinky back and forth between open hands and patting a ball against the shoulder. There were two conditions, each one comprising four trials. In the playful condition, the demonstrator displayed a playful, positive affect throughout the demonstration (as in experiment 1), while in the neutral condition, a different female demonstrator (of the same age and same ethnicity) showed neutral affect throughout the demonstration (see Fig. 2). The presentation of the video stimuli was arranged in two fixed random orders, which were counterbalanced across participants. For the purpose of maintaining attention and engagement, videos were interspersed with filler stimuli, which consisted of four short clips featuring popular cartoon characters such as Elmo and Teletubbies.

Participants were seated in a comfortable chair, 60 cm from the computer monitor in front of a small table, on which the materials necessary for the imitation of each trial were placed before the beginning of each trial. The objects and their arrangement were the same as those displayed in the video. No explicit direction was given, and participants’ spontaneous behavior with the objects in response to the demonstration was video-recorded for later coding.

Coding criteria for the imitation performance were the same as in experiment 1, with a total score calculated by summing the performance scores for each item and converting the sum into a percentage score. Coding was conducted using the videos by two research assistants who were blind to group membership and study hypotheses. Interrater reliability between the two research assistants for the coding of performance data was calculated on 20 % of the entire data set. The intraclass correlation was .98.

During observation of the video stimuli, participants’ eye-movements were recorded using the eye-tracker system and analyzed using frame-by-frame defined areas of interest using Tobii Studio analysis software. Fixation criteria were set to Tobii Studio defaults of a 30-pixel dispersion threshold for 100 ms. The two regions of interest included in the analyses were the model’s face and her action.

Eye-tracker calibration was controlled by Tobii Studio software. A five-point calibration and validation procedure was used, with calibrations being signaled as “valid” by the software when all five points showed good fit in the computed mapping for both eyes. The procedure was repeated until the five points were properly calibrated for each eye.

Five participants in the ASD group and two participants in the WS group were excluded due to inability to achieve calibration (one participant in each group) or equipment failure during the experiment.

##### Results

**Fig. 3:**
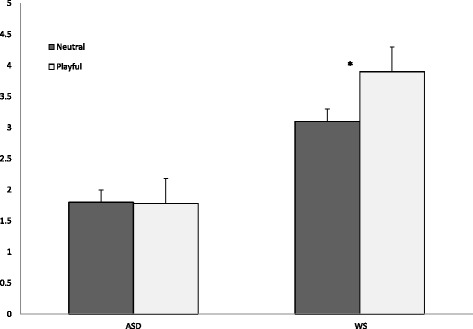
Average duration of fixations to the model’s face (experiment 2) *p = .005

First, participants’ visual attention to the model’s face (quantified in terms of average duration of fixations to the face region) was analyzed with a 2 (group) × 2 (condition) ANOVA. There was a main effect of the group (*F* (1, 48) = 12.61, *p* = .001, *η*_p_2 = .20), condition (*F* (1, 48) = 5.03, *p* < .05, *η*_p_2 = .09), and a significant group × condition interaction (*F* (1, 48) = 5.77, *p* < .05. *η*_p_2 = .10). As illustrated in Fig. 3, the children with ASD looked significantly less at the model’s face than those with WS. Further, pair-wise comparisons showed that while participants in the WS group increased their attention to the model’s face in the socially engaging condition compared with the neutral condition (adjusted *p* [Bonferroni] = .005, *η*_p_2 = .15), this was not the case in the ASD group (adjusted *p* = .89, *η*_p_2 = .00).

Secondly, with regard to participants’ visual attention to the model’s actions (quantified in terms of average duration of fixations to the action region), there was no effect of the group (*F* (1, 48) = 2.94, *p* = .09, *η*_p_2 = .05), condition (*F* (1, 48) = 1.82, *p* = .18, *η*_p_2 = .03) or group × condition interaction (*F* (1, 48) = .12, *p* = .56. *η*_p_2 = .00), suggesting that both groups attended to the actions for similar durations across conditions.

Finally, we tested participants’ imitation performance. The main effects of the group (*F* (1, 52) = .37, *p* = .05, *η*_p_2 = .07) and condition (*F* (1, 52) = 6.38, *p* = .01, *η*_p_2 = .10) were significant, but there was no group × condition interaction, (*F* (1, 52) = .26, *p* = .61. *η*_p_2 = .00), suggesting that both groups imitated more often in response to the playful compared to the neutral model presented on the video screen, although the children with WS were more likely to imitate both models. As shown in Table 3, imitation performance in the WS group was 52.63 (SD = 40.73) in the neutral condition, and 64.37 (SD = 34.67) in the playful condition. In the ASD, it was 35.00 (SD = 40.31) in the neutral condition, and 42.85 (SD = 35.62) in the playful condition. While the majority of children across groups spontaneously imitated at least one action, a small percentage of children in the ASD group (13 %) did not engage in any imitative behavior.

For both groups, attention to the model across conditions was unrelated to age, cognitive, adaptive and language functioning. In ASD, imitation in response to the playful model was negatively correlated with the ADOS social affect scores (*r* = −.61, *p* < .001) and positively correlated with the VABS socialization scores (*r* = .46, *p* < .01), MSEL developmental quotient (*r* = .36, *p* < .05), and VABS adaptive behavior composite score (*r* = .36, *p* < .05), suggesting that children in this group with more advanced social, cognitive, and adaptive skills were more likely to imitate the playful model. Similarly, imitation in response to the neutral model was negatively correlated to the ADOS social affect scores in the ASD group (*r* = −.48, *p* < .005). As in experiment 1, the only significant correlation in the WS group was between imitation performance across conditions and chronological age (*r* = .81, *p* < .001 for imitation of the playful model and *r* = .83, *p* < .001 for the imitation of the neutral model).

#### Experiment 3

The aim in experiment 3 was to investigate participants’ visual attention and spontaneous imitation in the context of an instrumental imitation task, i.e., a task where imitation served a purely instrumental function (retrieving a desired object from a container). As the social affiliation components were minimized, we expected imitation performance and visual attention to the model to be unaffected by the differences in social engagement across the ASD and WS groups.

##### Methods

**Fig. 4:**
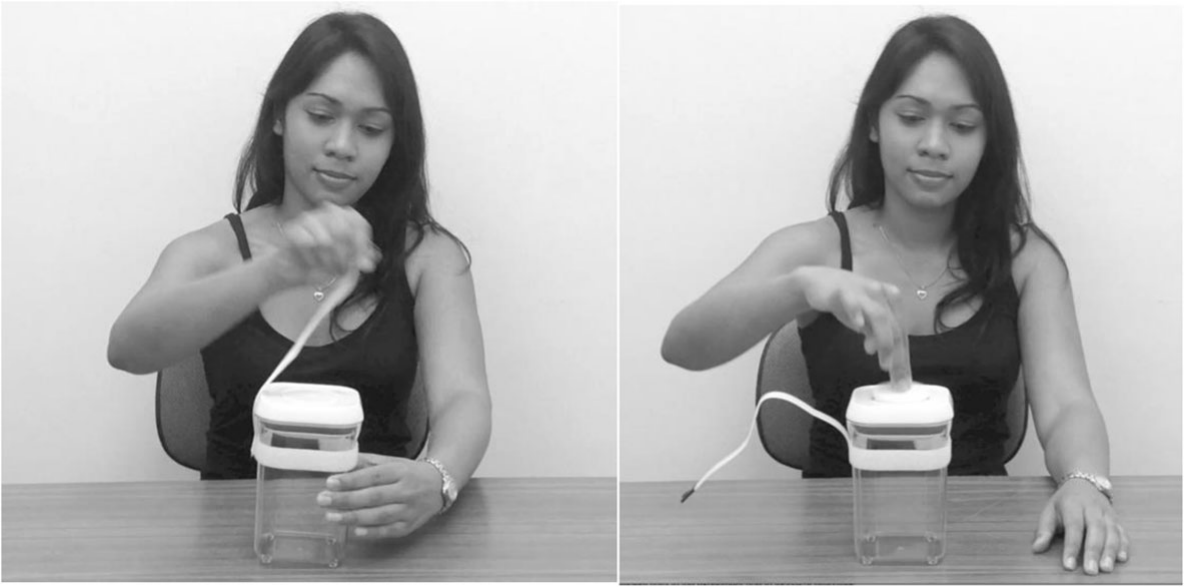
Example of video stimulus in experiment 3

Participants were seated in a comfortable chair, 60 cm from the eye-tracking computer as in experiment 2, in front of a small table. A series of nine 8-s videos were presented on the monitor in two different fixed random orders. A new female actor was used in all videos. During each video demonstration, the same actor showed how to open a container that could only be opened using a specific and novel sequence of two steps. For example, as illustrated in Fig. 4, one container required removal of a piece of Velcro followed by depression of a button on the lid before the lid could be opened.

Each trial involved a different container, and after the demonstration of each video, the same container shown in the video was given to the child. Inside the container was a toy that the child was motivated by during a brief warm-up free play episode prior to the experiment. Examples of toys used for the task were Thomas the Tank Engine and a Peppa Pig puppet. No instruction was given, and participants’ spontaneous behavior with the container in response to the demonstration was video-recorded for later coding. The only way to open the container in order to retrieve the toy was to follow the procedures demonstrated in the videos. The model did not display any social or emotional behavior. We therefore reasoned that performance in this task would reflect the instrumental function of imitation (i.e., imitating in order to achieve an instrumental goal, rather than to connect with the model).

The coding procedure involved simple yes/no (1/0) for imitating each action involved in the trial. A total imitation score was obtained for each participant by calculating the proportion of imitated actions out of the total imitation opportunities (i.e., the demonstrated actions presented in each of the videos). Coding was conducted by two research assistants blind to group membership and study hypotheses. Interrater reliability (intraclass correlation) between the two research assistants, calculated on 20 % of the entire data set, was .96.

During observation of the video stimuli, participants’ eye-movements were recorded using the eye-tracker system and analyzed using frame-by-frame defined areas of interest using Tobii Studio analysis software. Fixation criteria were set to Tobii Studio defaults of a 30-pixel dispersion threshold for 100 ms. The two regions of interest included in the analyses were the model’s face and her action. Two participants in the ASD group were excluded due to equipment failure during the experiment.

##### Results

As evident in Tables 2 and 3, there were no group differences in imitation performance (*t*(1, 54) = −.74, *p* = .42, Cohen’s *d* = −.20), in visual attention to the model’s face (*t*(1, 54) = −.01, *p* = .98, Cohen’s *d* = .00), or her actions (*t*(1, 54) = −1.65, *p* = .10, Cohen’s *d* = −.47). All children imitated at least one action, except for three children in the ASD group (8 %) and one in the WS group (4 %).

**Table 2:** Experiment 3, average duration of fixations to the areas of interest

Area of interest	Group		*t* test	*p* value	Cohen’s *d*
	ASD	WS			
Model’s face	1.36 (.83)	1.36 (.77)	−.01	.98	.00
Model’s action	3.71 (1.57)	4.39 (1.29)	−1.65	.10	−.47

*ASD* autism spectrum disorder, *WS* Williams syndrome

**Table 3:** Summary of learning performance across experiments

Experiment	Learning source	Learning function	Learning performance^a^—M(SD)		Group differences *p* value
			ASD	WS	
1	Neutral model—live	Social	19.09 (23.23)	29.86 (28.16)	.01^b^
	Playful model—live	Social	23.61 (23.67)	46.52 (23.40)	
2	Neutral model—video-recorded	Social	35.00 (40.31)	52.63 (40.73)	.61^b^
	Playful model—video-recorded	Social	42.85 (35.62)	64.37 (34.67)	
3	Neutral model—video-recorded	Instrumental	55.55 (32.23)	62.77 (33.87)	.42^c^
4	Own trials and errors	Instrumental	11.28 (28.46)	21.95 (21.55)	.20^c^

^a^Learning performance indicates average proportion of imitated actions in experiments 1, 2, and 3, and average improvement over time from first to last object presentation (expressed in a percentage score) in experiment 4

^b^2 (group; ASD, WS) × 2 (condition; neutral, playful) ANOVA group × condition interaction

^c^Between-group *t* test

Across groups, attention to the model was unrelated to age, cognitive, adaptive, and social functioning. In both ASD and WS groups, imitative performance was positively correlated with the VABS socialization scores (*r* = .45, *p* = .01 in ASD and *r* = .56, *p* < .01 in WS), MSEL developmental quotient (*r* = .53, *p* = .001 in ASD and *r* = .51, *p* < .05 in WS), and VABS adaptive behavior composite scores (*r* = .44, *p* = .01 in ASD and *r* = .66, *p* = .001 in WS). Additionally, in the ASD group, imitative performance was negatively associated with the ADOS social affect (*r* = -.32, *p* = .05) and positively associated with language as assessed through the MSEL (*r* = .40, *p* < .05 across receptive and expressive subscales). Additionally, in the WS group, there was a correlation between imitation performance and chronological age (*r* = .71, *p* < .001). As in the previous experiments, performance was unrelated to motor skills across groups.

#### Experiment 4

The rationale for experiment 4 was to investigate participants’ ability to learn from the consequences of their own actions via trial and error (non-social learning). As this task did not involve any social-processing demand, we expected performance to be unaffected by the differences in social engagement that distinguish ASD from WS.

##### Methods

Participants sat on a mat while they were presented with toy materials that could be only operated properly using one of four possible actions. There were three different sets of toys, including (1) a transparent plastic bag containing a toy with the four sides marked with different colors (blue, yellow, red, green); (2) a “pound-a-ball” toy with a squeaky hammer and four colored balls that drop through a maze when hit by the child; and (3) a drawing task where participants were presented with four colored markers and a blank paper. In each task, there was only one color that indicated the correct response: (a) only the yellow side opened the bag containing the motivating toy; (b) only the red ball could be hit with the hammer to drop through the maze; and (c) only one colored marker worked for the drawing task. No specific instruction was given. After children succeeded in operating the toy through trial and error (for example, opening the transparent bag from the yellow side after trying to open it from each side), the experimenter took the material for each task out of the child’s view, and then re-presented it to the child twice, to determine whether they learned from their own actions. We reasoned that if children learned from their own trial and error actions, then they would show more errors when attempting the task the first time compared to the second and third presentation. The child’s behavior in response to each object was video-recorded for later coding.

Performance scores were based on the difference in number of errors (i.e., number of incorrect items acted upon before identifying the correct one) occurring during the first and following presentations of the object, with a reduction in the number of errors indicating evidence of learning. The results from each trial were averaged to produce a total score. As the experimental procedure was based on the assumption that the child would be motivated to retrieve/operate the toy, trials were considered invalid if the child did not engage in any attempt to retrieve/operate the toys. This resulted in the exclusion of six participants in the WS group and four in the ASD group. Coding was conducted by a trained research assistant blind to group membership and study hypotheses. Interrater reliability between the first author and the research assistant was calculated on 20 % of the entire data set. ICC was .86.

##### Results

**Fig. 5:**
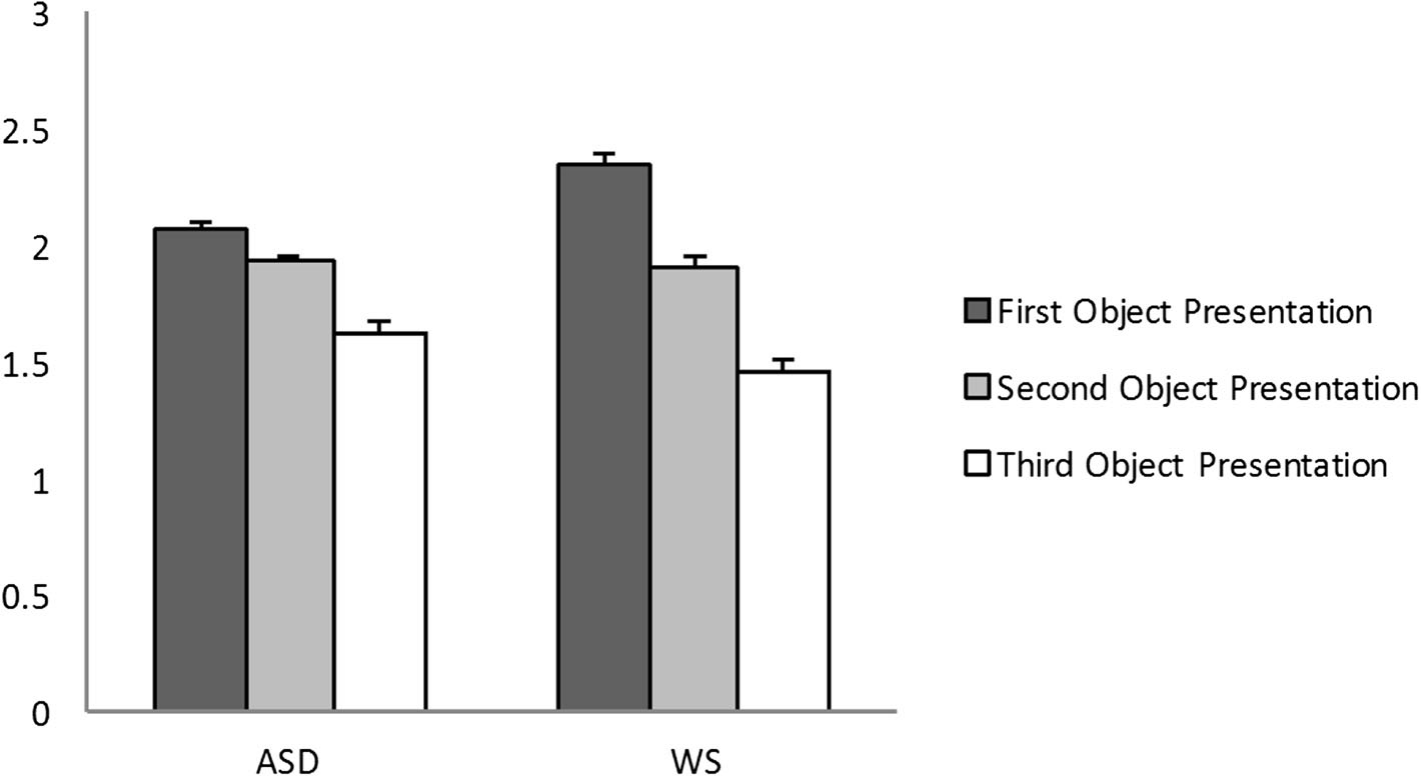
Number of errors in the first, second, and third object presentation in experiment 4

As illustrated in Fig. 5, participants in both groups appeared to make fewer errors in the second and third presentation of the object compared to the first presentation. To analyze group differences across presentations, participants’ performance scores (number of errors) in the first and second object presentation were submitted to a 2 (group) × 2 (condition—first and second object presentation) ANOVA. There was no main effect of the group (*F* (1, 44) = .13, *p* = .71, *η*_p_2 = .00) or group × condition interaction (*F* (1, 44) = 2.18, *p* = .14, *η*_p_2 = .04). The main effect of the condition was significant (*F* (1, 44) = 12.26, *p* = .001, *η*_p_2 = .21), indicating that all participants, irrespective to diagnostic group, made fewer mistakes in the second compared to the first attempt to operate the toy.

Similarly, participants’ performance scores in the second and third object presentation were submitted to a 2 (group) × 2 (condition—second and third object presentation) ANOVA. We found no main effect of group (*F* (1, 44) = .21, *p* = .65, *η*_p_2 = .00) nor group × condition interaction (*F* (1, 44) = .21, *p* = .64, *η*_p_2 = .00). The main effect of the condition was again significant (*F* (1, 44) = 6.13, *p* = .01, *η*_p_2 = .12), indicating that participants across both groups made fewer mistakes in the third compared to the second attempt to operate the toy.

Finally, participants’ performance scores in the first and third object presentation were submitted to a 2 (group) × 2 (condition—first and third object presentation) ANOVA. There was no main effect of the group (*F* (1, 44) = .08, *p* = .77, *η*_p_2 = .00) or group × condition interaction (*F* (1, 44) = 1.63, *p* = .20, *η*_p_2 = .03); however, the main effect of the condition was significant (*F* (1, 44) = 15.85, *p* < .01, *η*_p_2 = .26), indicating that all participants made fewer mistakes in the third compared to the first attempt to operate the toy irrespective of diagnostic group (see Table 3).

Overall, as illustrated in Fig. 5, these results suggest that the two groups were equally improving their performance with each attempt to operate the toy. In both ASD and WS groups, we found no significant correlations between performance in this task and cognitive, adaptive, social and motor functioning, and chronological age.

## Discussion

In this study, we measured how preschoolers with ASD and WS learn novel behaviors (1) from the results of their own actions (non-social learning), (2) from observing others’ actions (social learning), and across situations in which learning behaviors served either an instrumental function or a social affiliation motive. Through these manipulations, we sought to understand how differences in social functioning constrain learning processes in two neurodevelopmental disorders characterized by opposing social motivation profiles and similar life-long learning difficulties. Our results highlight several novel insights into the contextual factors modulating imitation and learning in preschoolers with ASD and WS.

### Social-emotional displays modulate social learning differently across ASD and WS

In experiment 1, children with WS imitated more frequently in response to a socially engaging compared to a neutral model. This pattern parallels reports in 24-month-old children documenting an increased propensity to imitate the outcomes of actions in response to socially engaging versus neutral (“aloof”) models [10, 11]. Therefore, it appears that the fascination that children with WS have for faces and emotional displays is beneficial, rather than being detrimental, to their social learning.

In contrast, results in experiment 1 indicate that children with ASD showed a lower propensity overall to imitate the model, and no modulation of imitation in response to the socially engaging (versus neutral) model during a live interaction. Additionally, we found that imitation performance was correlated with severity of symptoms of ASD within the social domain, as well as parent-reported socialization skills. These data are consistent with previous studies suggesting that children with ASD, as a consequence of their social impairments, might not register the social and communicative cues that facilitate imitative learning in children without ASD [27, 44]. However, the results from experiment 2 were inconsistent with our prediction, as children with ASD, like those with WS, imitated more often in response to the socially engaging versus neutral model when video-recorded demonstrations were used (although they were still showing a lower propensity overall to imitate the model). One possible explanation is that children with ASD have more difficulty with the complex array of signals that are present during a live demonstration, or perhaps the processing of relevant cues is facilitated for children with ASD by the simplified, “noiseless” nature of video-recorded stimuli [45]. However, more research is needed to substantiate these alternative explanations.

Interestingly, we found a consistent association across tasks between chronological age and imitation performance in the WS group but not in the ASD group, suggesting that while imitation in children with WS follows a developmental trajectory of increasing imitation proficiency over time, this might not be the case in ASD.

### Social-emotional displays modulate visual attention in WS but not in ASD

The eye-tracking paradigm used in experiment 2 enabled us to measure how visual attention to the demonstration was affected by the model’s social-emotional displays. We found that children with WS, but not those with ASD, increased their attention to the model’s face in the socially engaging condition compared to the neutral condition. These results are consistent with previous studies documenting a prolonged attention to the face in WS [46–48]. While it has been suggested that an increased focus on the face in WS might translate into a reduced attention to other relevant features (e.g., [38]), children with WS imitated more often in the social engagement condition across tasks, again suggesting that responsivity to facial social-emotional cues has a beneficial, rather than detrimental effect on imitative learning. Conversely, we found that visual attention was not modulated by the model’s social-emotional signals in the ASD group. This finding is consistent with previous studies pointing to reduced responsivity to social and emotional signals in ASD, which might contribute to core impairments in social learning [49, 50].

### When social learning serves an instrumental function, children with ASD and WS do not differ in imitative performance and visual attention to the model

Our results suggest that the opposing social phenotypes of ASD and WS do not translate into syndrome-specific differences in the instrumental function of social learning, neither with respect to attention to the model nor with respect to imitation performance. Both groups succeeded in learning the sequence of actions needed to obtain a motivating object to a comparable level, demonstrating similar imitation skills in situations where learning, rather than social affiliation, was instrumental to achieve a tangible goal. These results are consistent with the findings reported by Ingersoll et al. [51], who showed that children with ASD did not differ from typically developing controls in imitating actions that were instrumental to obtain a desired outcome. Taken together, these findings add to accumulating evidence that imitation difficulties in ASD may become more discernible in tasks involving social motivation components [30, 52].

Surprisingly, attention to the model in this task was similar in ASD and WS, a finding that contrasts with previous studies reporting that visual attention to faces is increased in WS and decreased in ASD [46, 47]. This finding suggests that differences in social attention across ASD and WS might be modulated by the specific context in which social information is presented. Thus, atypical attention to faces, rather than being an immutable inherent characteristic of these disorders, might be linked to the contextual factors of the social versus instrumental nature of the models’ actions and goals [53].

### Children with ASD and those with WS show similar abilities to learn from their own actions

Another aim in the current study was to explore whether the distinctive social profiles that characterize children with ASD and WS extend to the non-social learning domain. We found that children with ASD and WS were able to learn at a comparable level from their own actions in a trial-and-error task. This result confirms the notion that in learning tasks where social components are minimized, the performance of individuals with ASD is comparable to that of peers without ASD matched for IQ and motor skills.

There were several limitations in the current study that should be acknowledged. First, we did not systematically manipulate the visual-motor complexity of the tasks, and therefore the impact of this critical component on learning could not be assessed here. Second, the lack of randomization of the sequential order of conditions may have affected performance. Third, the potential bias introduced by multiple testing locations including the participants’ homes should be considered as a potential limitation, although results presented in the study remain unaltered when the three participants tested at home are removed from the analyses. Furthermore, while there was a non-significant trend toward the WS group being slightly older and more impaired, the different pattern of correlations between learning performance across experiments and chronological age, motor skills, and language suggest these differences may not have influenced the current findings. Another limitation concerns the lack of a mental age-matched control group of typically developing children. Finally, in order to make the experimental paradigm suitable for younger and severely affected children with ASD and WS and avoid fatigue and non-compliance, it was necessary to limit the number of trials and the verbal, cognitive and motor demands in each task.

Despite these limitations, our cross-syndrome approach and the novel experimental paradigms used in the current study should also be viewed as strengths. First, extreme care was taken in developing tasks that did not require “test-taking skills” such as understanding of verbal instructions and attention to relevant features of the task, which are often impaired in young children with ASD and WS. Additionally, the absence of explicit instructions enabled us to measure spontaneous, rather than “on the demand” imitative learning, and to avoid the possibility that social learning components (learning from the experimenter’s instructions and demonstration) were introduced in the task intended to measure non-social learning.

## Conclusions

In conclusion, we found that young children with ASD and WS showed similar abilities to learn new behaviors through active experience (non-social learning) and through observation of others (social learning) in response to situations that served instrumental purposes. However, the two groups responded differently to learning situations that involved social-affiliative motives, with children with WS, but not those with ASD, increasing their attention and imitative learning performance when the model acted in a socially engaging manner during a live interaction. Increased responsivity to social-emotional signals appears to have facilitative, rather than negative effects on imitative learning in children with WS. Conversely, lack of social responsivity might affect social learning in ASD, at least in the context of live interactions. These findings point to the context-dependent nature of imitative learning, suggesting that processes of knowledge acquisition should be investigated within a framework that takes into account both the social and instrumental motives underlying learning.

## Abbreviations

ASD: Autism spectrum disorder
WS: Williams syndrome
VABS: Vineland adaptive behavior scale
ADOS: Autism diagnostic observation schedule
MSEL: Mullen scales of early learning
